# Successful conservative treatment for massive uterine bleeding with non-septic disseminated intravascular coagulation after termination of early pregnancy in a woman with huge adenomyosis: case report

**DOI:** 10.1186/s12905-020-00924-8

**Published:** 2020-03-19

**Authors:** Fuminori Kimura, Akimasa Takahashi, Jun Kitazawa, Fumi Yoshino, Daisuke Katsura, Tsukuru Amano, Takashi Murakami

**Affiliations:** Department of Obstetrics and Gynecology, Shiga University of Medical Science, Seta Tsukinowa-cho, Otsu, Shiga 520-2192 Japan

**Keywords:** Adenomyosis, Case report, Disseminated intravascular coagulation, Termination of pregnancy, abortion

## Abstract

**Background:**

Adenomyosis is a benign gynecological condition in which endometrial tissue or endometrial-like tissue develops within the uterine myometrium. Few cases of disseminated intravascular coagulation has been reported in the patients with adenomyosis. Although hysterectomy is indicated for refractory massive uterine bleeding in the patients with advanced uterine adenomyosis, conservative treatment is often desired in women in the late reproductive age. Recently such cases are increasing due to the social trend of late marriage.

**Case presentation:**

A 37-year-old woman with huge adenomyosis, gravida 2 para 0, was referred to our hospital to terminate her pregnancy. Acute, non-septic, disseminated intravascular coagulation (DIC) developed after early pregnancy was terminated in a woman with huge adenomyosis. Massive bleeding and DIC occurred 3 days after the dilatation and curettage. There was no evidence of infection as the cause of the DIC, because neither bacteria nor endotoxin could be detected in her blood, and antithrombin 3 (AT3), which would be expected to decrease in septic patients, was not decreased. Hemorrhage in the adenomyotic tissue after the termination presumably developed inflammation, with numerous microthrombi and necrosis in the adenomyotic tissue, which subsequently promoted coagulation and fibrinolysis, leading to the onset of massive uterine bleeding and DIC. Although severe hyperfibrinolysis is observed in peripheral blood, the fibrinolysis state in the uterine myometrium is considered to be even more severe. The newly formed clots for hemostasis under the uterine mucosa could be removed due to the excessive activation of fibrinolytic system happened in the adjacent myometrium, leading to the onset of massive uterine bleeding. Massive bleeding and DIC resolved quickly after the patient was treated with nafamostat mesilate, which is effective for both excessive coagulation and fibrinolysis.

**Conclusions:**

Adenomyosis could cause massive bleeding and DIC when pregnancy is terminated. Massive bleeding was considered to occur because the excessive fibrinolysis system inside adenomyosis affected the adjacent endometrium. Before considering hysterectomy to control refractory uterine bleeding, nafamostat mesilate should be considered as one option, thinking the pathophysiology of the massive bleeding due to uterine adenomyosis.

## Background

Disseminated intravascular coagulation (DIC) is a coagulopathy that can occur in a large number of clinical conditions, including malignant tumors and obstetrical events [1–5]. However, few cases of DIC have been reported in benign diseases, especially intrauterine diseases [6–8]. Adenomyosis is a benign gynecological condition in which endometrial tissue or endometrial-like tissue develops within the uterine myometrium [9–11]. A case of non-septic DIC that occurred after termination of early pregnancy in a woman with huge adenomyosis is presented. The DIC occurred during the period when levels of pregnancy-related hormones and substances were diminishing. The clinical course strongly suggested that the massive bleeding was related to the occurrence of excessive fibrinolysis. Although hysterectomy is indicated for massive uterine bleeding in patients with advanced uterine adenomyosis, conservative treatment is often desired in women in the reproductive age. Recently, such cases have been increasing due to the social trend of late marriage. The administration of nafamostat mesilate, which is effective for both excessive coagulation and fibrinolysis, should be considered as one option when massive bleeding caused by excessive fibrinolysis due to huge adenomyosis occurs in patients who want to preserve their uterus.

## Case presentation

A 37-year-old woman, gravida 2, para 0, was referred to our hospital for termination of early pregnancy. She suffered from huge adenomyosis and had been treated with a GnRH agonist for 15 months in total. She also had a history of transfusion due to severe uterine hemorrhage when a miscarriage occurred at the age of 33 years.

**Fig. 1:**
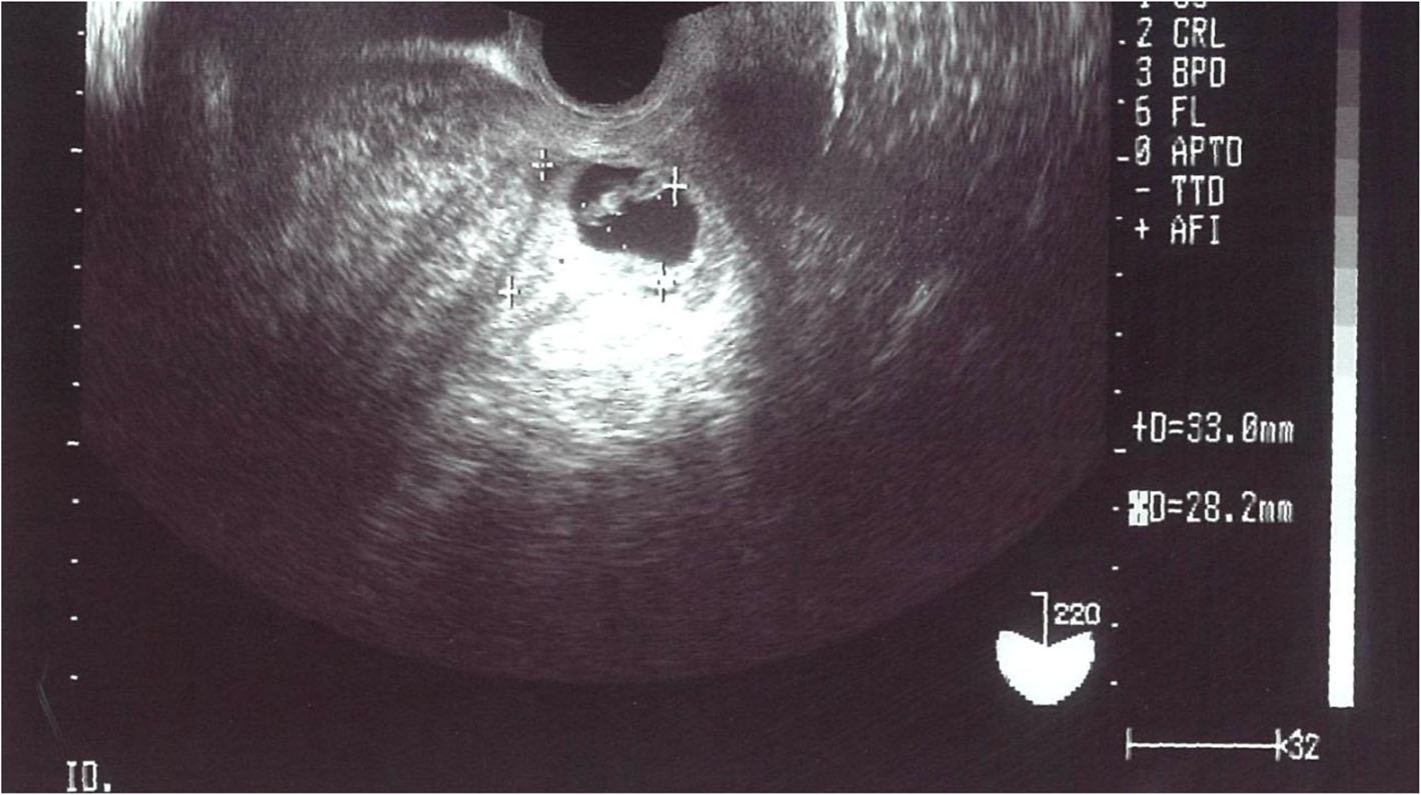
Ultrasound image of patient’s uterus at the first visit. The uterus was more than 10 cm in the thickness, and heterogeneous echogenicity could be diffusely detected throughout the myometrium. The uterine cavity was not enlarged and was rather narrow. It contained a 3 cm of gestational sac with fetal heart movement

**Fig. 2:**
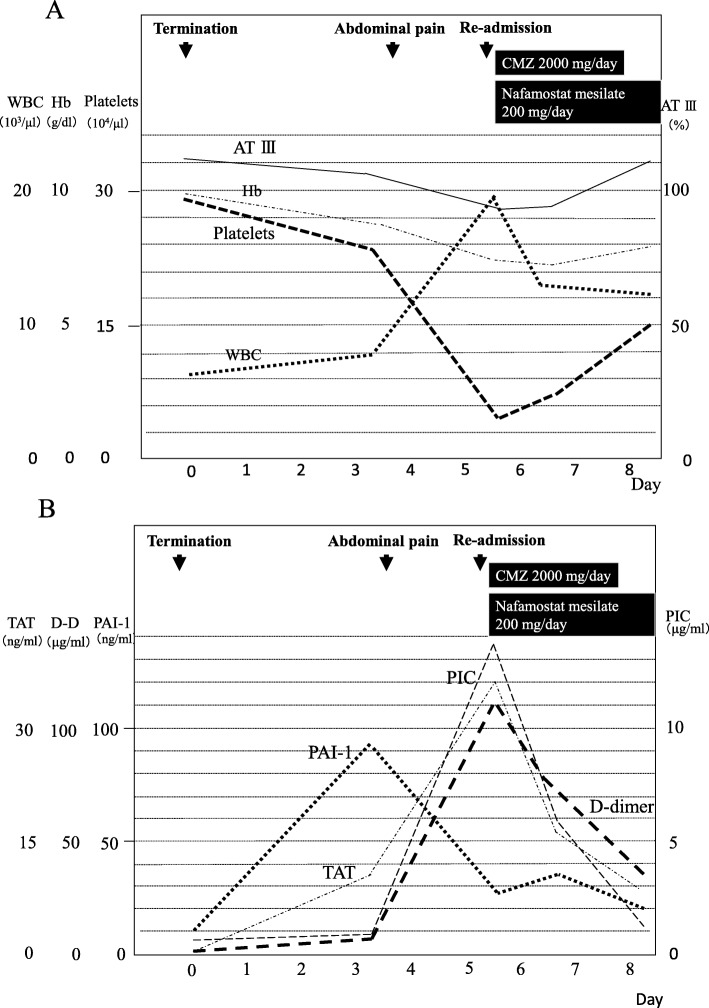
**a**. Changes in hemoglobin levels, white blood cell counts, platelets counts and levels of antithrombin 3. At 3 days after the procedure, the hemoglobin (Hb) level and the platelet count was slightly decreased from the initial data. The white blood cell (WBC) count and the level of antithrombin 3 (AT3) was in normal range. At fifth day of post procedure, the Hb level was decreased and the platelet count was markedly decreased. The WBC count was markedly increased although the level of AT3 was still in normal range. After the treatment of nafamostat mesylate and cefmetazole, the data were recovered. **b**. Changes in levels of D-dimer, thrombin antithrombin3 complex, plasminogen activator inhibitor-1 and plasminα2-plasmin inhibitor complex. The level of plasminogen activator inhibitor-1 (PAI-1) and the level of thrombin antithrombin 3 complex (TAT) was increased by the third day after the procedure, while the level of D-dimer and the level of plasminα2-plasmin inhibitor complex (PIC) were in normal range. The levels of D-dimer, TAT and PIC increased markedly on the fifth day although the level of PAI-1 started to decease. Treatment with nafamostat mesilate led to rapid improvement of the levels of D-dimer, TAT and PIC

On initial ultrasonography examination, the total thickness of her uterus was found to be over 10 cm, and extensive distribution of heterogeneous echogenicity was diffusely detected in both the posterior and anterior walls, as well as at the fundus. A 3-cm gestational sac with fetal heart movement, compatible with a gestational age of 6 weeks, was also detected in the uterine cavity (Fig. 1). In Japan, women have right to terminate pregnancy in early stage because of the socio-economic reason. In the present case, the woman was not married at that time and wanted to terminate the pregnancy due to financial reason. She was admitted, with dilatation and curettage performed gently under ultrasonographic guidance after the length of the uterine cavity was estimated to be 8.5 cm with a sonde, which was not very enlarged. There was little bleeding during the procedure. To prevent infection, 1 g of cefmetazole (CMZ) was administered intravenously twice in the perioperative period. The patient recovered well and was discharged the next day. When the patient was reviewed in the morning 3 days after the procedure, she had slight uterine bleeding with no pain or fever. Laboratory tests were as follows: white blood cells (WBCs) 8.0 × 10^3^ (3.3 × 10^3^–9.0 × 10^3^/μl), hemoglobin (Hb) 8.8 (11.5–15.0 g/dl), platelets 24 × 10^4^ (12 × 10^4^ – 40 × 10^4^ /μl), C-reactive protein (CRP) 0.9 mg/dl (< 0.5 mg/dl), anti-thrombin 3 (AT3) 108 (80–130%), thrombin antithrombin III complex (TAT) 10.9 (< 3.0 ng/ml), D-dimer 6.1 (< 1.0 μg/ml), plasminogen activator inhibitor-1 (PAI-1) 91.4 (< 50 ng/ml), and plasmin α2-plasmin inhibitor complex (PIC) 0.8 (0.4–0.8 μg/ml) (Fig. 2a, b). However, she developed abdominal pain that night, and the symptom gradually worsened. The amount of uterine bleeding also increased dramatically. She consulted our hospital again 5 days after the procedure because of severe abdominal pain and massive uterine bleeding. Her body temperature was 37.1 °C. There was marked tenderness of the uterus, and massive uterine bleeding continued. Her WBC count was 19.2 × 10^3^/μl, and her platelet count was decreased to 5.3 × 10^4^ /μl. Other laboratory tests were as follows: Hb 7.5 g/dl, CRP 24.1 (< 0.5 mg/dl), AT3 87%, TAT 36.0 ng/ml, D dimer 111.1 μg/ml, PAI-1 27.3 ng/ml, and PIC 13.7 μg/ml (Fig. 2a, b). These findings strongly suggested that the patient was developing DIC. Endotoxin was not detected in her blood, and no bacterial colonies grew in peripheral blood and uterine blood cultures. The *Chlamydia trachomatis* antibody titer was also not elevated. She was re-admitted to hospital and treated with nafamostat mesilate (200 mg/day) for 60 h, which has effects against both excessive coagulation and fibrinolysis, and cefmetazole (2 g/day) for 2 days. A decrease in bleeding was observed in a few hours. There was rapid improvement of her symptoms of abdominal pain and uterine bleeding and the laboratory data (Fig. 2a, b). Without surgical intervention, the patient was discharged on the fourth days after the second admission.

## Discussion and conclusions

In the present case, the clinical course and laboratory data strongly suggested that the DIC was unrelated to infection. First, symptoms of abdominal pain usually persist for several days after the start of treatment in the case of severe uterine infection, but the symptoms diminished rapidly after the administration of cefmetazole and nafamostat mesilate in the present case. Second, no colonies grew or were detected on peripheral blood and uterine blood cultures, and endotoxin was neither detected in her blood. Third, the serum level of AT3 was not decreased so much, and PAI-1 was not increased when the patient complained of abdominal ache, whereas these parameters should be markedly decreased and markedly elevated, respectively, in a case of septic DIC. Accordingly, mechanisms other than infection are thought to have been responsible for her DIC. Thus, adenomyosis was thought to be the main cause of DIC after the termination of early pregnancy, although it is difficult to reliably deny other causes.

Adenomyosis is a benign condition, but it is known to cause hypermenorrhea, dysmenorrhea, and infertility [9–11]. Hypermenorrhea may be due to increased area of the uterine cavity, disturbance of uterine contraction, and hemorrhage from the adenomyosis tissue to the uterine cavity [12]. Inflammation and hemorrhage are known as potential causes of dysfunctional coagulation and fibrinolysis [13–15]. Recently, the occurrence of DIC in a patient with adenomyosis during menstruation was reported [7]. This report suggested that local hemorrhage inside the adenomyosis and subsequent thrombosis formation contributed to the development of acute DIC. We recently reported activation of the fibrinolysis system during menstruation and its relationship to microthrombi inside adenomyosis in patients with large adenomyosis, and we suggested that intramural hemorrhage in the adenomyosis lesion blocked vessels, leading to multiple microthrombi and ischemic damage to the myometrium, resulting in activation of the fibrinolysis system [16]. Although severe hyperfibrinolysis is observed in peripheral blood, we speculate the fibrinolysis state in the uterine myometrium is considered to be even more severe. The newly formed clot for hemostasis under the uterine mucosa could be removed due to the excess fibrinolytic substance(s) from the adjacent myometrium, leading to the onset of massive uterine bleeding. Difficulty of the treatment of uterine adenomyosis might be that uterine adenomyosis produces fibrinolytic substances, that directly effect on the endometrium that lies on adjacent adenomyosis. Moreover, a similar adenomyosis case of acute DIC that developed after dilation and curettage was reported [8]. The clinical course improved after treatment with tranexamic acid, blood transfusions, and subtotal hysterectomy. The authors also stated that activation of the coagulation system, microthrombus formation, myometrial necrosis, exhaustion of coagulation factors, and hyperfibrinolysis might play crucial roles in the development of DIC [8].

In the present case, the increase of sex steroid hormones due to pregnancy could have affected the adenomyosis tissue and resulted in the production of more microthrombi than usual menstruation. The patient did not complain of abdominal pain until 3 days after the procedure. In addition, 3 days after the procedure, AT3 was in the normal range. The Hb level and platelet count were slightly decreased, and PAI-1 was increased to 91.4 ng/ml. TAT and D dimer increased slightly to 10.9 ng/ml and 6.1 μg/ml, respectively, while PIC was unchanged. PAI-1 inhibits the serine protease tissue plasminogen activator (tPA) and is thus an inhibitor of fibrinolysis. As it inhibited activation of blood fibrinolysis, DIC might not occur at that time. Five days after the procedure, the Hb level and platelet count were decreased, and PAI-1 was decreased to 27.3 ng/ml in normal range. However, TAT was increased to 36.0 ng/ml, and D dimer and PIC were increased to 111.1 μg/ml and 13.7 μg/ml, respectively, although AT3 was still in the normal range. Based on these data, the patient could be diagnosed as developing DIC with both excess coagulation and fibrinolysis. We thought that a large number of thrombi had already formed 3 days after the procedure. Considering the changes of PIC and D dimer, fibrinolysis was excessive with increased coagulation after 3 days of the procedure. Therefore, we thought that a large number of microthrombi had formed inside the adenomyotic tissue around 3 days after the procedure, which subsequently led to the rapid onset of DIC with excessive fibrinolysis. Since severe fibrinolysis dysfunction was confirmed in the peripheral blood, it was presumed that the fibrinolysis dysfunction inside the uterus was further enhanced. For this reason, massive uterine bleeding occurred, and it is thought that a rapid decrease in the Hb level and a decrease in the platelet count were observed.

Four cases of cerebral infarcts associated with adenomyosis have been reported [17]. Interestingly, 2 of the 4 patients had systemic embolism, and another patient had thrombi in the brachiocephalic trunk and left subclavian artery. The levels of coagulation markers were reported to be elevated in the acute phase. The authors speculated that patients with adenomyosis might be potentially at risk of developing infarcts associated with hypercoagulability related to menstruation-related coagulopathy or increased tissue factors. Moreover, a case of uterine infarction inside uterine adenomyosis following biochemical pregnancy was reported [18]. Necrotic myometrium due to focal uterine infarction was suspected based on pelvic magnetic resonance imaging.

In the present case, nafamostat mesilate was administered because the coagulation function was still activated, and nafamostat mesilate has effects against both excessive coagulation and fibrinolysis to prevent the development of infarcts [19, 20]. Administration of heparins alone to fibrinolytic DIC (DIC with a strong fibrinolytic activation) rather promotes bleeding [21–23]. Both gabexate mesilate (FOY) and nafamostat mesylate are synthetic serine protease inhibitors and exhibit antithrombin-independent anticoagulant activity [22–26]. However, FOY does not have strong effect to inhibit fibrinolytic activity. Nafamostat mesylate is characterized by a strong effect of inhibiting not only the activation of coagulation but also the activation of fibrinolysis [24–26]. For this reason, it is an extremely effective therapeutic agent for fibrinolytic DIC. To treat excessive fibrinolysis, we could choose to administer a drug with only antifibrinolytic action, such as tranexamic acid. However, we thought that it might increase the risk of developing infarcts. Nafamostat mesilate can be expected to suppress new redundant production of microthrombi inside adenomyotic tissue. In our view, a drug affecting both coagulation and fibrinolysis should be considered when massive bleeding occurs in patients with huge adenomyosis. Moreover, nafamostat mesylate has a short half-life of 5–8 min and we can quickly switch treatment strategy to surgery if it is judged to be ineffective [21, 27].

She was very grateful that she saved her uterus as she was unmarried and desired to get a child in future.

To the best of our knowledge, this is the first report of successful conservative treatment of acute non septic DIC after termination of early pregnancy in a woman with adenomyosis. The clinical course strongly suggested that bleeding inside the adenomyotic tissue promoted excessive coagulation and fibrinolysis, which was then related to the development of DIC. Although severe hyperfibrinolysis is observed in peripheral blood, the fibrinolysis state in the myometrium is considered to be even worse. The newly formed clot for hemostasis under the uterine mucosa could be removed due to the excess fibrinolytic system in the adjacent myometrium and could cause massive bleeding. This case serves as a warning that adenomyosis might induce DIC with excessive fibrinolysis after termination of pregnancy as a trigger and the usefulness of nafamostat mesilate to treat it in patients with huge adenomyosis who want to preserve their uterus. Before considering hysterectomy to control refractory uterine bleeding, nafamostat mesilate should be considered as one option, thinking the pathophysiology of the massive bleeding due to uterine adenomyosis. Conservative treatment can be considered in the case of “women who wants to conserve her fertility”.

## Data Availability

We can provide the raw data. The datasets used and/or analysed during the current study available from the corresponding author on reasonable request.

## Abbreviations

AT3: Anti thrombin 3
CMZ: Cefmetazole
CRP: C-reactive protein
DIC: Disseminated intravascular coagulation
FOY: Gabexate mesylate
Hb: Hemoglobin
PAI-1: Plasminogen activator inhibitor-1
PIC: Plasmin α2-plasmin inhibitor complex
TAT: Thrombin antithrombinIIIcomplex
tPA: serine protease tissue plasminogen activator
WBC: White blood cell

## References

[1] Feinstein DI (2015). Disseminated intravascular coagulation in patients with solid tumors. Oncology.

[2] Levi M (2016). Management of cancer-associated disseminated intravascular coagulation. Thromb Res.

[3] Franchini M, Di Minno MN, Coppola A (2010). Disseminated intravascular coagulation in hematologic malignancies. Semin Thromb Hemost.

[4] Cunningham FG, Nelson DB (2015). Disseminated intravascular coagulation syndromes in obstetrics. Obstet Gynecol.

[5] Erez O, Mastrolia SA, Thachil J (2015). Disseminated intravascular coagulation in pregnancy: insights in pathophysiology, diagnosis and management. Am J Obstet Gynecol.

[6] Antovic J, Bakic M, Milicevic R, Gojkovic G, Blombäck M (2001). Activation of the coagulation system occurs within rather than outside cutaneous haemangiomas. Acta Paediatr.

[7] Nakamura Y, Kawamura N, Ishiko O, Ogita S (2002). Acute disseminated intravascular coagulation developed during menstruation in an adenomyosis patient. Arch Gynecol Obstet.

[8] Zhang J, Xiao X, Luo F, Shi G, He Y, Yao Y, Xu L (2013). Acute disseminated intravascular coagulation developed after dilation and curettage in an adenomyosis patient: a case report. Blood Coagul Fibrinolysis.

[9] García-Solares J, Donnez J, Donnez O, Dolmans MM (2018). Pathogenesis of uterine adenomyosis: invagination or metaplasia?. Fertil Steril.

[10] Pontis A, D’Alterio MN, Pirarba S, de Angelis C, Tinelli R, Angioni S (2016). Adenomyosis: a systematic review of medical treatment. Gynecol Endocrinol.

[11] Struble J, Reid S, Bedaiwy MA (2016). Adenomyosis: a clinical review of a challenging gynecologic condition. J Minim Invasive Gynecol.

[12] Devlieger R, D'Hooghe T, Timmerman D (2003). Uterine adenomyosis in the infertility clinic. Hum Reprod Update.

[13] Krychtiuk KA, Kastl SP, Speidl WS, Wojta J (2013). Inflammation and coagulation in atherosclerosis. Hamostaseologie.

[14] Simmons J, Pittet JF (2015). The coagulopathy of acute sepsis. Curr Opin Anaesthesiol.

[15] Reikerås O, Borgen P (2014). Activation of markers of inflammation, coagulation and fibrinolysis in musculoskeletal trauma. PLoS One.

[16] Yamanaka A, Kimura F, Yoshida T, Kita N, Takahashi K, Kushima R (2016). Dysfunctional coagulation and fibrinolysis systems due to adenomyosis is a possible cause of thrombosis and menorrhagia. Eur J Obstet Gynecol Reprod Biol.

[17] Yamashiro K, Tanaka R, Nishioka K, Ueno Y, Shimura H, Okuma Y (2012). Cerebral infarcts associated with adenomyosis among middle-aged women. J Stroke Cerebrovasc Dis.

[18] Lee JY, Hwang KR, Won KH, Lee DY, Jeon HW, Moon MH (2014). Uterine infarction in a patient with uterine adenomyosis following biochemical pregnancy. Clin Exp Reprod Med.

[19] Shimada M, Matsumata T, Shirabe K, Kamakura T, Taketomi A, Sugimachi K (1994). Effect of nafamostat mesilate on coagulation and fibrinolysis in hepatic resection. J Am Coll Surg.

[20] Sundaram S, Gikakis N, Hack CE, Niewiarowski S, Edmunds LH, Koneti Rao A (1996). Nafamostat mesilate, a broad spectrum protease inhibitor, modulates platelet, neutrophil and contact activation in simulated extracorporeal circulation. Thromb Haemost.

[21] Choi JY, Kang YJ, Jang HM, Jung HY, Cho JH, Park SH (2015). Nafamostat Mesilate as an Anticoagulant During Continuous Renal Replacement Therapy in Patients With High Bleeding Risk: A Randomized Clinical Trial. Medicine (Baltimore).

[22] Taneichi A, Fujiwara H, Mizoguchi Y, Machida S, Nonaka H, Takei Y (2014). Disseminated intravascular coagulopathy caused by uterine leiomyoma with sarcoma-like findings on magnetic resonance imaging. Case Rep Obstet Gynecol.

[23] Mukaiyama H, Shionoya S, Ikezawa T, Kamiya T, Hamaguchi M, Saito H (1987). Abdominal aortic aneurysm complicated with chronic disseminated intravascular coagulopathy: a case of surgical treatment. J Vasc Surg.

[24] Asakura H (2014). Classifying types of disseminated intravascular coagulation: clinical and animal models. J Intensive Care.

[25] Fujii S, Hitomi Y (1981). New synthetic inhibitors of C1r, C1 esterase, thrombin, plasmin, kallikrein and trypsin. Biochim Biophys Acta.

[26] Aoyama T, Ino Y, Ozeki M, Oda M, Sato T, Koshiyama Y (1984). Pharmacological studies of FUT-175, nafamstat mesilate. I. Inhibition of protease activity in in vitro and in vivo experiments. Jpn J Pharmacol.

[27] Shimokawa Miyama T, Yoshioka C, Minami K, Okawa T, Hiraoka H, Itamoto K (2010). Nafamostat mesilate is not appropriate as an anticoagulant during continuous renal replacement therapy in dogs. J Vet Med Sci.

